# Translation, cross-cultural adaptation and validation of the Slovenian version of Harris Hip Score

**DOI:** 10.1186/s12955-020-01592-w

**Published:** 2020-10-08

**Authors:** Petra Josipović, Metka Moharič, Dea Salamon

**Affiliations:** 1 Univerza v Ljubljani Medicinska Fakulteta, Vrazov trg 2, Ljubljana, Slovenia; 2 Univerzitetni rehabilitacijski institut SOČA, Linhartova 51, Ljubljana, Slovenia; 3 Univerza na Primorskem Fakulteta za vede o zdravju, Polje 42, Izola, Slovenia; 4 Pula, Croatia

**Keywords:** Hip, Slovenian, Harris hip score, Translation, Validation

## Abstract

**Introduction:**

The Harris Hip Score is the most widely used outcome measure for the assessment of hip pathologies. An official Slovenian version has not been culturally adapted and validated. The aim of this study was to create a Slovenian valid and reliable version of the HHS.

**Materials and method:**

The HHS was translated and modified in Slovenian. The measurement properties of the Slovenian HHS were tested in 42 patients suffering from different hip pathologies. Reliability, responsiveness, construct validity, convergent/divergent validity and content validity of the Slovenian version of the HHS were tested.

**Results:**

Only minor adaptation was required in the translation process. The internal consistency of the HHS expressed by Cronbach’s alpha was 0.94. The test–retest reliability expressed by the intraclass correlation coefficient was 0.983. The correlations of the HHS scale with the WOMAC scale (r = − 0.877) and the VAS scale (r = − 0.717) were statistically significant. The highest correlation between the HHS and SF-36 was with the General Health dimension (r = 0.61). while the lowest correlation was with the SF-36 Mental Health dimension (r = 0.43). MDC95% was 10.1. No floor or ceiling effects were found.

**Conclusion:**

Slovenian version of HHS seems to has an acceptable level of reliability and validity. Slovenian HHS is short, comprehensible and easy to administer and interpret.

***Trial registration*:**

Approved by the Slovenian National Medical Ethics Committee (0120-46/2019/19).

## Introduction

Many outcome measures have been developed for the assessment of hip pathologies, such as the Oxford Hip Score, Nonarthritic Hip Score, Hip and Groin Outcome Score, International Hip Outcome Tool, Hip Outcome Score, Hip Dysfunction and Osteoarthritis Score, and Merle d’Aubigné and Postel score [1–6]. The Harris Hip Score (HHS) is one of the most widely used health related quality of life measures for the assessment of hip pathology [7]. The HHS was developed for the assessment of the results of hip surgery and evaluation of various hip disabilities in an adult population [8]. The HHS is administered by a physician or physiotherapist and presents a scale with the maximum of 100 points, including evaluation of pain, function, deformity and motion [8]. The pain domain measures pain severity and its effect on activities and need for pain medication. The function domain is divided into daily activities (stair use, using public transportation, sitting, and managing shoes and socks) and gait (limp, support needed, and walking distance). The deformity domains observe hip flexion, adduction, internal rotation, and extremity length discrepancy while the range of motion (ROM) domain asses hip ROM [8]. The range of motion item consists of 6 motions that are graded based on the arc of motion possible. Each range of motion gradation is assigned an index factor and a maximum possible value, which are used to calculate arc of motion points [8]. These points are added and multiplied by 0.05 to receive the total points for range of motion. The total score is calculated by summing the scores for the 4 domains [8, 9]. The score is covering pain (1 item, 0–44 points), function (7 items, 0–47 points), absence of deformity (1 item, 4 points), and range of motion (2 items, 5 points). A total score below 70 points is considered a poor result, 70 to 80 reasonable, 80 to 90 good and 90 to 100 excellent [10].

Outcomes such as quality of life related to health, functional capacity, pain and satisfaction scales have been emphasized as they enable the analysis of the state of health and manifestations of disease in individuals’ lives [1–10]. Malchau et al. [11] showed the HHS to be a reliable and valid measure of hip function. Several studies have used the HHS as a patient self-report questionnaire [11, 12]. Mahomed et al. [12] made comparison study of patient self-report HHS with surgeon assessment. Overall, the self-report and surgeon-assessed HHS showed excellent concordance. The results of similar studies support the use of the HHS as a self-report instrument [13]. In contrast, Lieberman et al. [14] found significant differences between patient self-report and physician evaluation of outcomes after total hip arthroplasty.

According to available sources in databases, the HHS has been translated, culturally adapted and validated on Italian [15], Turkish [16], Arabic [17] and Portuguese [18] language. Construct and criterion validity of the HHS Italian Version were confirmed by satisfactory values of Spearman s Rho for correlation between specific domains of HHS and Western Ontario and Mac Master Osteoarthritis Index (WOMAC) and the Short Form Health Survey (SF-36) scores [15]. Interobserver and test–retest reliabilities obtained values of 0.996 and 0.975 respectively. Cronbach s alpha for internal consistency was 0.816 [15]. The Turkish version of the HHS showed sufficient internal consistency (Cronbach’s alpha, 0.70) and test–retest reliability (ICC = 0.91) [16]. The correlation coefficients between the HHS, the WOMAC and the Oxford Hip Score were 0.64 and 0.89 respectively [16]. Internal consistency of the Arabic version of HHS was estimated by calculating Cronbach's alpha in three different occasions. For each of the three administrations of instruments, the internal consistency, estimated using Cronbach's alpha, was very good or excellent—α_1_ = 0.92, α_2_ = 0.91, and α_3_ = 0.90. The Arabic version of the HHS showed good test–retest reliability (ICC = 0.76) (95% CI 0.44–0.88) [17]. The Brazilian version of the HHS was translated and culturally adapted but not yet validated. A further study is currently underway to evaluate the reliability and validity of the culturally adapted Brazilian version [18].

In spite the fact that HHS is the most widely used outcome measure for the assessment of hip pathologies, an official Slovenian version has not been cultural adopted and validated. When there is an assessment protocol described and validated in another language, it is necessary to standardize the cross-cultural equivalence methodology in the language to be used for this protocol to be employed [19, 20].

The aim of this study was to provide a reliable Slovenian version of the Harris Hip Score. The authors aimed to translate the HHS and adapt it to the Slovenian culture, following the guidelines for validation and cross-cultural adaptation stated by Guillemin et al. [21].

Considering the psychometric information of the HHS and currently existing validation studies protocol, authors determined and chose the WOMAC, the SF-36 and Visual Analogue Scale (VAS) to be compared to the HHS.

## Methods

The HHS was translated and validated on Slovenian language as a part of a doctoral thesis research project, which aimed to develop and explore effects of the medical device named Hip traction and Vibration device for hip pain in elderly patients with primary symptomatic coxarthrosis. The method of translation and cultural adaptation of the Harris Hip Score used the criteria described by Guillemin et al. [21], which involved four stages: initial translation; back-translation; examination of the versions with preparation of a consensus version; and commented pre-test with development of the final version.

### Translation and cross-cultural adaptation

Two well-qualified Slovenian translators, fluent in English language were responsible for the literary and conceptual translation of the HHS. First informed translator was a physiotherapist and the second one was a professional literary translator. Both translators’ mother tongue was Slovene and both were fluent in English. Both translations were compared and reviewed by a specially established group for this.

task consisting of one medical doctor who is specialist in physical medicine and rehabilitation; a two PhD. students of Biomedicine (Medical Faculty in Ljubljana) and one occupational therapist. The group highlighted any conceptual errors or inconsistencies in the translations in order to establish a single preliminary draft, synthesized from the separate forward translations. The backward translation of the HHS was carried out by a professional linguist with a university degree in English who had never seen the original English version of the questionnaire. At the end, all corrections were collected and a single affordable translation was created.

Six patients with diagnosis of coxarthrosis (N = 3) and hip fracture (N = 3) have tested the pre-final version to ensure understanding the purpose and meaning of each question to provide the final Slovenian version of the HHS. Patients expressed their opinions on used wording, understandability, interpretation, and cultural relevance of the translation. The final version of the Slovenian version of the HHS was approved after the final review.

### Participants

Patients were regular residents of the nursing home “Lucija” in Portorož where study was performed. The inclusion criteria for the patients were: Coxarthrosis, Femoral fracture, Hip arthroplasty, Osteoporosis, Avascular necrosis, Hip pain, Congenital dislocation of hip, Hip effusion, Muscle tear, Edema of femoral head, Acetabular cystic lesion. All participants who passed eligibility criteria were asked to read and sign an informed consent form that had been approved by the Slovenian National Medical Ethics Committee (0120–46/2019/19).

Out of 180 elderly patients with different hip pathologies (Coxarthrosis, Femoral fracture, Hip arthroplasty, Osteoporosis, Hip pain) were initially considered for inclusion, 85 were eligible to enter the study, while 31 did not meet inclusion criteria. Amid eligible patients 12 refused to participate in the study. Participants were excluded from the study due to the inability to: to cooperate, understand and fulfill the questionnaires, understand the Slovenian language, have other inabilities to participate in the study (i.e., medical conditions, being alcohol or substance dependent, or current alcohol or substance abuse, cardiac or other medical instability, immobilized, fractured, having active malignancy, and mental illness). Finally, 42 elderly patients with different hip pathologies were enrolled into the study.

Minimal sample size (SS_min_) was calculated via free G*Power 3.1.9.4 software (Faul, Kiel, Germany). G*Power is a tool to compute statistical power analyses for many different t tests, F tests, χ^2^ tests, z tests and some exact tests. G*Power can also be used to compute effect sizes and to display graphically the results of power analyses [22]. A priori correlation power analysis and sample size calculations were performed by assuming the population correlation alternative hypothesis—(pH_1_ = 0.70) and determining the population correlation assuming null hypothesis—(pH_0_ = 0.70). Furthermore, calculated effect size was 0.5, α error probability was 0.05 and power (1-β err prob) was 0.95. Minimal sample size required for validation was 42.

The authors determined and chose the Western Ontario and McMaster Universities Arthritis Index (WOMAC), the Short form-36 Health survey (SF-36) and Visual Analogue Scale (VAS) to be compared to the HHS. We specifically determined: the reliability, the responsiveness, the validity by correlation with the WOMAC [23], the Short form-36 Health survey [24] and VAS [25], which are culturally adapted and validated on Slovenian language. The patients were asked to complete the Slovenian version of the HHS, the WOMAC, the SF-36 and VAS. Ten days after first assessment, patients were asked again to complete Slovenian version of HHS to determine the test–retest reliability. One physiotherapist and one occupational therapist provided assistance in reading, writing, and explanation, if requested. The study was performed between September 2019 and March 2020.

The HHS is a clinician-based, joint-specific assessment tool and requires the health-care professional to grade the patient’s pain (44 points), mobility and walking (47 points), range of motion (5 points), and absence of deformities (4 points). Each question is answered using a Likert scale with an overall score ranging from 0 (extreme symptoms) to 100 (no symptoms). A total HHS of ˂70 points is considered poor result, 70 to 80 is fair, 80 to 90 is good, and the 90 to 100 is excellent [26].

The WOMAC is a self-administered, disease-specific measure that contains subscales for pain, stiffness, and physical function [27]. The original global score is calculated as the sum of the scores for each subscale. Scores range from 0 to 20 (pain), 0 to 8 (stiffness), and 0 to 68 (function) [27]. The higher the score, the worse the health state.

The SF-36 comprises eight scaled scores; each scale is directly transformed into a scale from 0 to 100 to identify the patient’s physical and mental state [28]. These eight sections are: physical functioning (PF); role limitations due to physical function (RP); bodily pain (BP); general health perceptions (GH); vitality (VH); social function (SF); emotional function (RE); and mental health (MH) [28, 29].

### Statistical analysis

Reliability, responsiveness, construct validity, convergent/divergent validity and content validity of the Slovenian version of the HHS were tested. Free Statistics Software, version 1.2.1 (Wessa P, Leuven, Belgium) was used for statistical analysis. Descriptive statistic was calculated (frequency counts and percentages for nominal variables, measures of central tendency and dispersion for continuous variables. The level of significance was p˂0.05. The Kolmogorov–Smirnov test was used for assessment of the normality of the distribution.

#### Reliability

Reliability consists in a measure of consistency, repeatability and agreement of experimental results [7, 20]. Internal consistency was assessed using Cronbach’s alpha (α) coefficient. It estimates coherence among each component of the test. It is a measure of the homogeneity of the questions within a questionnaire [15, 30]. An α coefficient of 0.70–0.95 was considered to be adequate [15, 16, 30]. Test–retest reliability requires two administrations of the instrument during a period of time when no change in the target concept has occurred [31] and represents a scale’s capability of giving consistent results [16, 31]. The intra-class correlation coefficient (ICC) was used to measure the test–retest reliability of the Slovenian version of HHS. Correlation values of r ≥ 0.40 were considered satisfactory, r ≥ 0.81–1.00 was excellent, 0.61–0.80 was very good, 0.41–0.60 was good, 0.21–0.40 was fair, and 0.00- 0.20 was poor [22]. Most of the studies consider ICC to be good when it ranges between 0.6 and 0.9 [15, 32] so we determined as criterion ICC > 0.65.

#### Responsiveness

Responsiveness refers to the ability of an outcome measure to detect change when it has truly occurred, either as the result of an intervention or over time. Responsiveness is reported using change (or difference) scores. The Standard error of measurement (SEM) is calculated as the SD of the scores × the square root of (1-ICC), using data from the first and second administrations of the Slovenian version of the HHS. The minimal detectable change (MDC) refers to the minimal amount of change that is within measurement error. The SEM was used to determine the MDC at the 95% limits of confidence (MDC95%) and was calculated as the SEM × 1.96 × the square root of 2 [16, 33].

#### Validity

Validity is the criterion whereby an outcome measure is tested for its ability to actually measure what it aims to measure [15, 33]. Pearson’s correlation coefficients and their 95% confidence intervals were calculated to assess construct validity and convergent/divergent validity. The construct validity of the Slovenian version of HHS was provided by determining its relationship with the WOMAC, the VAS and the physical component summary (PCS) of the SF-36. The PF, RP, and PCS domains of the SF-36 were used to assess the convergent validity. The divergent validity of the Slovenian HHS was provided by determining its relationships with the MH, RE, and mental component score (MCS) domains of the SF36 [15–17, 32, 33]. Content validity was assessed by the distribution and occurrence of ceiling and floor effects [1, 23]. Floor and ceiling effects of the HHS at the first and second administrations were assessed by calculating the proportion of the patients scoring the minimal (score of 0) or maximal (score of 100) scores relative the total number of patients [15, 32, 33]. Floor and ceiling effects were relevant if > 30% of the patients had a floor and ceiling effect [16, 32, 33]. Less than 15% of results achieving minimum or maximum values means good content validity [15, 33].

## Results

### Translation and Cross-cultural adaptation

The aforementioned research group consisting of multidisciplinary members compared the original and the back-translated versions of HHS in order to identify, discuss and resolve the semantic and conceptual discrepancies. Subsequently, the differences between the original and the translated versions were addressed in a group discussion. Afterwards, each group member suggested the possible solution to resolve the addressed semantic and conceptual discrepancies. Each group member than ranked the suggestions from best to worst (not allowing ties). If needed, anonymous voting method on paper was used in purpose to choose the most appropriate solution out of the best ranked solutions. The solutions with the highest total ranking and votes were accepted in the final version. During the translation process of original HHS into the Slovenian version, few corrections were made as follows: In domain pain, the term aspirin was considered as a synonym for “Protibolečinsko zdravilo” (pain-killers). In domain function, we came across on a term “blocks walked” which represented the distance walked in American culture. In Slovenian culture, the distance is expressed by the time or meters so we adopted the terms “Nekaj ulic (30 min)” (several streets, 30 min) instead of term “six blocks” and “2–3 ulice (10–15 min)” (2–3 streets, 10–15 min) instead of term “two or three blocks”. After testing of the pre-final version of the Slovenian version of HHS on six patients and final review by the multidisciplinary group, no difficulties were found. Pre-final version of Slovenian HHS was accepted as the final version.

### Statistical analysis

#### Descriptive analysis

The study involved 42 participants (9 male and 33 female). The median age of all subjects was 84 (range 63–99 years). The median age of the male participants was 81 (range 65—87) and the median age of the female participants was 84 (range 63–99). Most participants, 50%, were diagnosed with coxarthrosis.

The median on the Slovenian HHS in the first administration was 65 (range 2–99), while the median in the second administration was 67 (range 6–100). The arithmetic mean in the first administration was 59.71 (SD = 27.85) and in the second administration was 62.31 (SD = 25.60). The median on the WOMAC scale was 35 (range 1–97.92) and the arithmetic mean was 34.42 (SD = 32.28). The median on the VAS scale was 3 (range 0–10) and the arithmetic mean was 3.37 (SD = 2.52).

The T-test for the dependent samples showed that there was a statistically significant difference between the arithmetic means of the HHS scale in the first and second administrations, with a higher result in the second administration. The effect size expressed by Cohen's D index was 0.40, which is a small to medium effect (Cohen's D index = arithmetic mean / standard deviation). Significant differences in arithmetic means between the first and second administration, and the small to medium effect of change expressed by the Cohen D index indicate on possible inherent bias.

The Kolmogorov–Smirnov test for normality of the distribution, showed that the HHS scores in the first and second administration did not deviate statistically significantly from the normal distribution. The same was for WOMAC scores while VAS scores did not have a normal distribution (Table 1). The distribution of VAS scores on the right is asymmetric because the sample was dominated by participants with a lower score on the scale. The skewness and kurtosis values for all variables are between − 2 and 2, which is considered acceptable for the use of parametric statistics [34].

**Table 1:** The Kolmogorov–Smirnov test for normality of the distribution

	Kolmogorov-Smirnov^a^ test			Skewness		Kurtosis	
	Statistic	*df*	Sig	Skewness	Std. Error	Kurtosis	Std. Error
HHS_1_overall	0.089	42	0.200*	− 0.550	0.365	− 0.533	0.717
HHS_2_overall	0.122	42	0.117	− 0.705	0.365	− 0.235	0.717
WOMAC	0.125	42	0.097	0.689	0.365	− 0.213	0.717
VAS	0.177	42	0.002	0.731	0.365	− 0.387	0.717

*This is a lower bound of the true significance

^a^Lilliefors Significance Correction

#### Reliability

Cronbach’s α of 0.94 for the Slovenian version of the HHS was above the level generally considered acceptable for research purposes (more than 0.70). The test–retest reliability of the HHS test was excellent, with an intraclass correlation coefficient of 0.983.

#### Responsiveness

Responsiveness is reported using change (or difference) scores. The SEM was used to determine the MDC at the 95% limits of confidence and was calculated as the SEM × 1.96 × the square root of 2 [16, 33]. The SEM of HHS was 3.6 and the MDC95% was 10.1.

#### Validity

The HHS scale was highly correlated with the WOMAC scale (r = − 0.877) and the VAS scale (r = − 0.717). Both correlations were statistically significant. Correlations of the HHS scale with all eight dimensions of the SF-36 scale were statistically significant. Correlations of the HHS scale with all eight dimensions of the SF-36 scale are shown in (Table 2).

**Table 2:** Correlations of the HHS with eight dimensions of the SF-36 scale

		PF	RP	VT	SF	RE	MH	GH	BP
HHS	Pearson r Correlation	0.498**	0.519**	0.482**	0.460**	0.444**	0.437**	0.616**	0.442**
	Sig. (2-tailed)	0.001	0.000	0.001	0.002	0.003	0.004	0.000	0.003
	N	42	42	42	42	42	42	42	42

^**^Correlation is significant at the 0.01 level (2-tailed)

The highest correlation was between the Slovenian HHS and the general health dimension (GH, r = 0.616) of SF-36. The lowest correlation was between Slovenian HHS and the mental health dimension (MH, r = 0.437) of SF-36. The correlation with the Physical Component Summary (PCS) of SF-36 (r = 0.687) was higher than the correlation with the Mental Component Summary (MCS) of SF-36 (r = 0.548).

The value of the effect size of Pearson r correlation varies between − 1 (a perfect negative correlation) to + 1 (a perfect positive correlation). The effect size is low if the value of r varies around 0.1, medium if r varies around 0.3, and large if r varies more than 0.5 [34], meaning that effect sizes for correlations of the Slovenian version of HHS with SF-36 domains vary from medium to large. Therefore, effect sizes for correlations of the Slovenian HHS with the WOMAC and VAS scales are large.

Floor and ceiling effect were calculated for the first and second administration of the HHS scale. No patient had a minimal result in either assessment. One patient (2.38%) scored the highest in the second HHS administration.

## Discussion

The cross-cultural adaptation strives to ensure consistency in the validity of content between the versions of the questionnaire (original and in the target language). Subtle differences in living habits in the different cultures might make an item from the questionnaire more or less difficult to understand, and may alter the psychometric and statistical properties of the tool [15–21]. The final version of the Slovenian HHS revealed no complications within comprehension. Furthermore, the Slovenian version of the HHS seems to has demonstrated acceptable levels of reliability and validity of evaluation of patient-reported outcomes of Slovenian-speaking individuals with a variety of hip pathologies. The internal consistency of the HHS expressed by Cronbach's alpha was 0.94 and was above the level generally considered acceptable for research purposes (more than 0.70). However, it is suggested that the value of Coefficient alpha should not be more than 0.95 as it means multicollinearity between the items and indicated that some items are redundant or a number of items asking the same question in slightly different ways [35]. Cronbach s alpha for internal consistency of Italian version of HHS was 0.816 [15] and 0.70 [16] for the Turkish version respectively.

The test–retest reliability of the Slovenian version of HHS seems to be excellent, with an ICC of 0.983, similar to the results of previously reported data [15–17]. Hinman et al. reported a lower test–retest reliability with an ICC value of 0.76 [36]. Studies of test–retest reliability for health-related Quality of Life instruments have used varying intervals between test administrations. The interval has ranged from 10 min to 1 month [37–43]. In the literature, the reported time intervals for estimation of test–retest reliability for the HHS was from 7–14 days [37, 44]. In short re-test intervals, patients can answer questions simply based on their memory of the first assessment while longer intervals can carry the risk of the spontaneous improvement of patient condition. In general, the length of time between repeat administrations of a patient-reported outcome measure should be relatively short (3–7 days) when the condition being measured is expected to change rapidly [36, 37, 44]. Participants in our study did not undergo interventions that would imply rapid changes in condition and 50% of them were diagnosed with coxarthrosis. This means that the hip conditions included such as coxarthrosis are not likely to change so significantly over a (1–2) weeks period, so regarding to that, authors have chosen time interval of 10 days based on similar studies [17, 37]. Marx et al. [37] reported no clinically or statistically significant difference between the measurement of test–retest reliability performed with a 2-day interval as compared with a 2-week interval for athletic patients with disorders in their study. 2- or 14-days’ time interval is considered short enough to prevent any change in patient’s disease, and long enough to avoid the patient remembering the answers given the first time [37]. The patients need not complete the second test on an exact date, but rather within this time frame [37]. There is a lack of evidence available to aid in the selection of the time interval between questionnaire administration for a study of test–retest reliability for health status instruments in patients with osteoarthritis [16, 37].

HHS responsiveness has been determined in a study of 335 THA. The effect size between preoperative and 6-months postoperative was excellent for pain (2.80) and function (1.72), but weak in the 2-years follow up, i.e., pain (0.15) and function (0.18) [38].

The SEM can be used to generate the MDC, which is the minimal amount of change in the score of an instrument that must occur in an individual in order to be sure that the change in score is not simply attributable to measurement error [34]. Çelik et al. [16], reported that MDC and SEM of Turkish HHS were 13.3 and 4.9 respectively. The SEM for the Slovenian version of HHS was 3.6 and the MDC95% was 10.1.

Considering the currently existing validation studies protocol, authors determined and chose the WOMAC, the SF-36 and Visual Analogue Scale (VAS) to be compared to the HHS (6–9, 15–17). The construct validity of the Slovenian version of HHS was provided by determining its relationship with the WOMAC, VAS and the PCS of the SF-36. The Slovenian version of HHS is highly correlated with the WOMAC scale (r = − 0.877), the VAS scale (r = − 0.717) and PCS domain (r = 0.687) of SF-36. The Turkish version of the HHS demonstrated a very good correlation with the WOMAC (r = 0.75 and 0.64, respectively) and a good correlation with the VAS score (r = 0.60) [16].

The convergent validity of the Slovenian HHS was provided by determining its relationships (correlations) with the SF-36 PF (r = 0.49), SF-36 PCS (r = 0.68) and SF36 RP (r = 0.51). The divergent validity of the Slovenian HHS was provided by determining its relationships with the SF-36 MH (r = 0.43), SF-36 RE (r = 0.44) and SF36 MCS (r = 0.54). As expected, the HHS was more strongly related to concurrent measures of physical function than to concurrent measures of mental function, similar to previous validation studies [15–17]. We found the highest correlation value between the HHS and SF-36 GH domain and the lowest correlation value between the HHS and MH domain (r = 0.43), as expected. The Turkish version of the HHS was most strongly related to the PF, PCS, and BP subdomains of the SF-36 (r = 0.72, 0.63, and 0.70, respectively). The weakest correlations between the Turkish HHS and SF-36 were seen in the MH and MCS subdomains (r = 0.10 and 0.14, respectively) [16]. Construct and criterion validity of the HHS Italian Version were confirmed by satisfactory values of Spearman s Rho for correlation between specific domains of HHS and WOMAC and the SF-36 scores [15].

No floor or ceiling effect was found in this study. No patient had a minimal result in either administration while only one patient (2.38%) scored the highest in the second HHS administration. This provides evidence for good content validity.

## Limitations

Limitations of this study include the fact that there was a heterogeneity of hip conditions with high proportion of patients with coxarthrosis (50%), which may not be representative of the general population. Out of 180, 42 elderly patients with different hip pathologies were enrolled into the study which represents very small sample size. A small sample size and a heterogeneity of hip conditions may have affected the reliability of an HSS instrument results because it may have led to a higher variability, which may have led to inherent biases (test–retest). The HHS has been re-administrated by two healthcare professionals which may have led to biases related to the administrator. Possible observer bias can also occur when the subject knows they are being examined. When a subject knows they are being observed, it can cause them to act differently from how they normally would, which could interfere with the experiment.

## Conclusion

The psychometric characteristics of the Slovenian version of the HHS seems to be satisfying. The test–retest reliability of the HHS seems to be excellent (ICC = 0.983). The Slovenian version of the HHS seems to be highly and statistically significantly correlated to the WOMAC scale, the SF-36 and the VAS scale as previously done validation studies have reported. Respect to acceptable levels of reliability and validity, we believe that Slovenian version of the HHS is sufficient to evaluate the state of a hip disease. The HHS is short, comprehensible and easy to administer and interpret. For future psychometric validation studies of the Slovenian version of the HHS, researchers need to consider enlargement of the sample size of highly compliant patients and minimization of risk for biases (e.g. re-administration of the instrument by the same healthcare professional).

## Supplementary information

**Additional file 1:** The final version of the Slovenian Harris Hip Score.

## Data Availability

The datasets used and analysed during the current study are available from the corresponding author on reasonable request. Some of data generated or analysed during this study are included in this published article [and its Additional file 1].
