# Supplementary material for: Translation, cross-cultural adaptation and validation of the Slovenian version of Harris Hip Score
Source: Health Qual Life Outcomes. 2020 Oct 8;18:335. doi: 10.1186/s12955-020-01592-w (PMC7545539; doi:10.1186/s12955-020-01592-w)
Supplement: Supplementary file 1 — Additional file 1: The final version of the Slovenian Harris Hip Score. [file 12955_2020_1592_MOESM1_ESM.pdf]

Harrisova kolčna lestvica (HHS)

Ime pacienta: _________________ Oboleli kolk: D L (obkroži)

Datum: :_____________________

1. del

Bolečina

☐ Je ni ali je ne opazim	+44
☐ Rahla, občasna, ne vpliva na izvedbo dejavnosti	+40
☐ Blaga bolečina brez vpliva na vsakodnevne dejavnosti. Ob nevsakdanjih aktivnostih se redko pojavi bolečina, ki potrebuje uporabo protibolečinskega zdravila	+30
☐ Zmerna, še znosna bolečina. Omejuje izvedbo vsakodnevnih dejavnosti in dela. Občasno je potrebno močnejše protibolečinsko zdravilo.	+20
☐ Močna bolečina, ki resno omejuje izvedbo dejavnosti/dela.	+10
☐ Popolnoma nemočen zaradi bolečine, nepomičen, vezan na posteljo	+0

Šepanje

☐ Ni	+11
☐ Rahlo	+8
☐ Zmerno	+5
☐ Hudo oziroma nezmožnost hoje	+0

Opora

☐ Ni potrebna	+11
☐ Palica/pohodne palice za daljšo hojo	+7
☐ Palica/pohodne palice večinoma	+5
☐ Ena bergla	+3
☐ Dve palici/pohodne palice	+2
☐ Dve bergli ali nezmožnost hoje	+0

Prehojena razdalja

☐ Neomejena	+11
☐ Nekaj ulic (30 minut)	+8
☐ 2-3 ulice (10-15 minut)	+5
☐ Samo po stanovanju	+2
☐ Samo na postelji oziroma na stolu	+0

Sedenje

☐ Brez težav na običajnem stolu za eno uro	+5
☐ Na visokem stolu za 30 minut	+3
☐ Nezmožen sedenja v stolu	+0

Uporaba javnega prevoza

☐ Da	+1
☐ Ne	+0

Stopnice

☐ Normalno brez uporabe ograje	+4
☐ Normalno z uporabo ograje	+2
☐ Na kakršen koli način	+1
☐ Nezmožen hoje po stopnicah	+0

Obuvanje nogavic in čevljev

☐ Brez težav	+4
☐ S težavo	+2
☐ Ni možno	+0

2. del

Ali ima pacient vse našteto? (vse da=4, manj od 4=0)

☐ da	manj kot 30˚ fiksirane fleksije<br>manj kot 10˚ fiksirane addukcije<br>manj kot 10 ˚fiksirane notranje rotacije in ekstenzije<br>razlika v dolžini udov manjša od 3,2 cm
☐ ne	

3. del.

Obseg gibljivosti (normalni obseg giba)
Fleksija: (140˚):_____________
Abdukcija (40˚):_____________
Addukcija (40˚):_____________
Zunanja rotacija (40˚):________
Notranja rotacija (40˚):________

Točkovanje:
Obseg giba:___________
Popolni obseg giba:____________
211˚-300˚ =5 točk
161 ˚ -210˚ =4 točke
101˚ -160˚ =3 točke
61˚ -100˚ =2 točki
31˚ -60˚ = 1 točka
0˚ -30˚ = 0 točke

Dosežene točke gibljivosti:_________

Skupne točke Harrisove lestvice kolka:______________točk
